# Packing up the genome

**DOI:** 10.7554/eLife.94128

**Published:** 2023-12-14

**Authors:** Bálint Kiss, Miklós Kellermayer

**Affiliations:** 1 Department of Biophysics and Radiation Biology, Semmelweis University Budapest Hungary

**Keywords:** genome packaging, bacteriophage, structural biology, viruses, packaging motor, Viruses

## Abstract

Nucleotide and force-dependent mechanisms control how the viral genome of lambda bacteriophage is inserted into capsids.

**Related research article** Rawson B, Ordyan M, Yang Q, Sippy J, Feiss M, Catalano CE, Smith DE. 2023. Regulation of phage lambda packaging motor-DNA interactions: Nucleotide independent and dependent gripping and friction. *eLife* **12**:RP91647. doi: 10.7554/eLife.91647.

In order to survive, viruses must be able to copy themselves to make more viral particles. They do this by passing their genetic material to a host cell that can replicate their DNA and produce the components required to assemble more viruses. While all viruses have evolved remarkable mechanisms to ensure the successful completion of their life cycle, viruses containing double-stranded DNA display some of the most ingenious molecular tricks nature has to offer.

Imagine double-stranded DNA as a stiff, pencil-thick piece of yarn the length of a football pitch that needs to be quickly threaded into a protein capsule the size of a football, known as the capsid. During an infection, the packaged DNA strands must then be rapidly ejected from the viral capsid so they can be transmitted to another host cell. How a linear chain, that also displays random conformations, passes through the narrow opening of the capsid during viral genome packaging or subsequent ejection puzzled scientists for decades (Kasianowicz et al., 2002). But little more than twenty years ago, the fascinating mechanical features of viral DNA packaging were discovered (Smith et al., 2001).

Packaging is accomplished by a motor enzyme composed of five circularly arranged oligomers made up of large subunits (known as TerL) and small subunits (TerS) that work together in a coordinated manner (Chemla et al., 2005; Rao and Feiss, 2015). The motor converts energy (generated by breaking down the nucleotide ATP into ADP and inorganic phosphate) into movements that can rapidly thread the entire viral genome into the capsid without ever needing to dissociate and rebind to the DNA (Schliwa and Woehlke, 2003).

Previous work on a virus that infects bacteria called T4 bacteriophage showed that DNA can partially slip out of the capsid during packaging (Ordyan et al., 2018). To prevent this from occurring, the motor has a grip mechanism that controls this process, and an end clamp that stops the viral DNA from completely exiting the capsid once packaging has started. Yet, how the different subunits of the motor contribute to these processes, and how these processes differ in structurally distinct viruses, remained unknown. Now, in eLife, Douglas Smith (University of California San Diego), Carlos Catalano (University of Colorado) and colleagues – including Brandon Rawson as first author – report experiments investigating the mechanical properties of the motor complex in another virus, known as lambda bacteriophage (Rawson et al., 2023).

During packaging, the lambda motor complex frequently transitions between gripping the viral DNA in place and loosening its grip, resulting in the DNA slipping out of the capsid. To better understand the molecular interactions behind this, Rawson et al. employed optical tweezers – a tool that uses focused laser beams to create gentle forces – to pull on the free end of a partially packaged DNA molecule and to measure the direction and magnitude of DNA displacement. These experiments were conducted in a microfluidic chamber so that the virus-DNA assembly could be exposed to different biochemical environments.

To assess the role of ATP in this transition between slipping and gripping, the motor complex was studied in a nucleotide-free state, in the presence of either ADP, or a version of ATP that is not hydrolyzed into ADP. Rawson et al. found that in the nucleotide-free state, rapid DNA slippage occurred, but friction between the DNA and the motor and an end-clamp mechanism prevented complete escape of the DNA molecule. At times, slippage was randomly halted by gripping stages (Figure 1A). In the ADP condition, the frequency and duration of the DNA-gripped state increased, leading to significantly reduced DNA slippage. DNA slippage was essentially halted in non-hydrolyzable ATP conditions, with the DNA being gripped almost continuously (Figure 1B).

**Figure 1.**
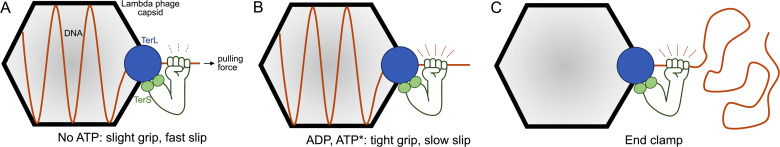
Mechanical control of genome packaging in the lambda bacteriophage. (**A**) The genome of the lambda bacteriophage virus consists of double stranded DNA (red) which is threaded into a viral capsid (hexagon; black line) by a complex made up of one large (TerL; blue) and two small (TerS; green) motor subunits. For simplicity, only one out of the five molecules in the motor complex is represented schematically. In the absence of the nucleotide ATP, the lambda-phage genome is under a slight grip that prevents the DNA molecule from escaping the capsid despite exposure to experimental pulling forces. Occasionally, however, rapid slipping bursts may occur. (**B**) In the presence of ADP or non-hydrolyzable ATP analogs (ATP*), the grip tightens and slipping bursts become slower and less frequent. (**C**) Complete departure of the DNA from the capsid is prevented by an end-clamp mechanism.

Applying force to the free end of the viral DNA using optical tweezers tilted the balance between slipping and gripping states towards slippage. This suggests that the motor of the lambda bacteriophage is regulated by both nucleotides and mechanical forces.

Previous studies have shown the motor complex of the T4 bacteriophage – which lacks the TerS subunits – does not grip when in a nucleotide-free state, in contrast to the lambda motor (Ordyan et al., 2018). This difference between lambda and T4 bacteriophages is likely related to the function of the TerS subunits, which may act as a sliding clamp that controls the level of friction between the motor proteins and DNA. The lambda bacteriophage does, however, display the end-clamp feature observed in T4 phages (Figure 1C), suggesting this is a general mechanism that ensures viral DNA packaging is efficient by avoiding complete genome release and the need to re-initiate the packaging process.

The tour-de-force experiments conducted by Rawson et al. are fundamental for understanding the ever-intricate mechanisms underlying the viral life cycle. A myriad of questions await exploration, however. For instance, how does the motor complex recognize the end of DNA to enable end-clamping? Are the gripping and slipping processes controlled by the same set of protein-DNA interactions? And does DNA ejection rely on similar mechanisms? It also remains to be seen whether these viral tricks could be manipulated to externally control and inhibit viral infections or employed in biotechnology. As questions continue to arise, the elegant viral phenomena will carry on fascinating scientists.
